# Methyl 2-{[3-(4,6-dimethoxy­pyrimidin-2-yl)ureido]sulfonyl­meth­yl}benzoate

**DOI:** 10.1107/S1600536808005011

**Published:** 2008-02-27

**Authors:** Jin-yun Xia, Fang-shi Li, Li-he Yin, Da-sheng Yu, Deng-yu Wu

**Affiliations:** a Department of Applied Chemistry, College of Science, Nanjing University of Technology, No. 5 Xinmofan Road, Nanjing 210009, People’s Republic of China

## Abstract

In the title compound, C_16_H_18_N_4_O_7_S, a synthetic sulfonyl­urea herbicide, there are intra­molecular N—H⋯N and C—H⋯O hydrogen bonds. Inter­molecular N—H⋯O and C—H⋯O hydrogen bonds form centrosymmetric dimers. The dihedral angle between the two rings is 50.00 (15)°.

## Related literature

For related literature, see: Kong *et al.* (1990 ▶); Lee *et al.* (2002 ▶); Sabadie (1996 ▶).
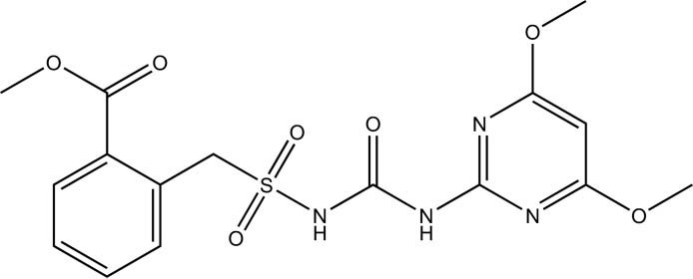


## Experimental

### 

#### Crystal data

C_16_H_18_N_4_O_7_S
*M* *_r_* = 410.41
Monoclinic, 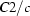

*a* = 33.831 (7) Å
*b* = 6.9020 (14) Å
*c* = 16.021 (3) Å
β = 104.48 (3)°
*V* = 3622.1 (13) Å^3^
*Z* = 8
Mo *K*α radiation
μ = 0.23 mm^−1^
*T* = 298 (2) K
0.40 × 0.20 × 0.10 mm

#### Data collection

Enraf–Nonius CAD-4 diffractometer
Absorption correction: ψ scan (North *et al.*, 1968 ▶) *T*_min_ = 0.914, *T*_max_ = 0.978
3325 measured reflections
3265 independent reflections
2421 reflections with *I* > 2σ(*I*)
*R*_int_ = 0.035
3 standard reflections every 200 reflections intensity decay: none

#### Refinement

*R*[*F* ^2^ > 2σ(*F* ^2^)] = 0.056
*wR*(*F* ^2^) = 0.156
*S* = 1.03
3265 reflections
253 parameters
H-atom parameters constrained
Δρ_max_ = 0.26 e Å^−3^
Δρ_min_ = −0.31 e Å^−3^

### 

Data collection: *CAD-4 Software* (Enraf–Nonius, 1989 ▶); cell refinement: *CAD-4 Software*; data reduction: *XCAD4* (Harms & Wocadlo, 1995 ▶); program(s) used to solve structure: *SHELXS97* (Sheldrick, 2008 ▶); program(s) used to refine structure: *SHELXL97* (Sheldrick, 2008 ▶); molecular graphics: *SHELXTL* (Sheldrick, 2008 ▶); software used to prepare material for publication: *SHELXL97*.

## Supplementary Material

Crystal structure: contains datablocks global, I. DOI: 10.1107/S1600536808005011/cf2185sup1.cif

Structure factors: contains datablocks I. DOI: 10.1107/S1600536808005011/cf2185Isup2.hkl

Additional supplementary materials:  crystallographic information; 3D view; checkCIF report

## Figures and Tables

**Table 1:** Hydrogen-bond geometry (Å, °)

*D*—H⋯*A*	*D*—H	H⋯*A*	*D*⋯*A*	*D*—H⋯*A*
N1—H1*A*⋯N3	0.86	1.94	2.648 (4)	138
N2—H2*A*⋯O5^i^	0.86	2.10	2.951 (3)	170
C9—H9*B*⋯O1	0.97	2.36	2.970 (4)	120
C15—H15*C*⋯O1^i^	0.96	2.43	3.068 (4)	124

Symmetry code: (i) 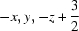.

**Fig. 1.**
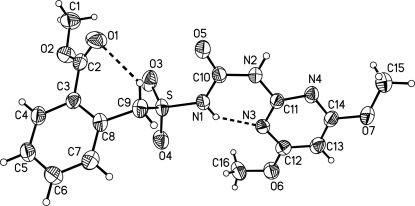
The molecular structure of (I). Displacement ellipsoids are drawn at the 50% probability level. Dashed lines indicate intramolecular hydrogen bonds.

**Fig. 2.**
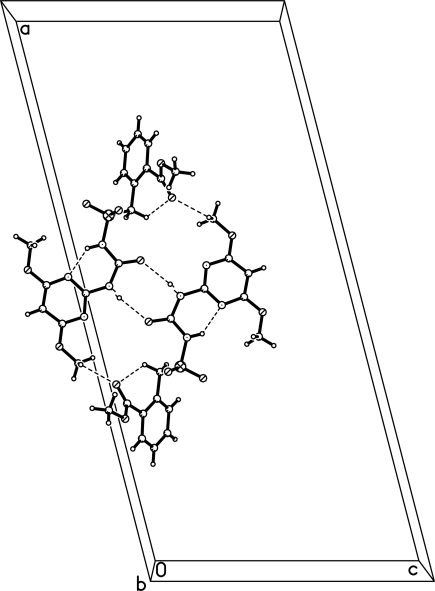
A hydrogen-bonded dimer. Dashed lines indicate hydrogen bonds.

## supplementary crystallographic information

### Comment

The title compound, bensulfuron-methyl, belongs to the class of systemic sulfonylurea herbicides inhibiting acetolactate synthase, a key enzyme in the biosynthesis of the branched-chain amino acids of target plants (Lee *et al.*, 2002). It is widely used in transplanted and direct-seeded rice fields to control most annual and perennial broadleaved weeds selectively (Sabadie, 1996).

We report here the crystal structure of the title compound, (I). The molecular structure of (I) is shown in Fig. 1. The dihedral angle between the C3–C8 and C11/N3/C12/C13/C14/N4 rings is 50.00 (15)°. There are intramolecular N1—H1A···N3 and C9—H9B···O1 hydrogen bonds (Fig. 1), and intermolecular N—H···O and C—H···O hydrogen bonds form centrosymmetric dimers (Fig. 2).

### Experimental

The title compound, (I), was prepared according to the literature method (Kong *et al.*, 1990).

Crystals suitable for X-ray analysis were obtained by dissolving (I) (0.2 g) in ethyl acetate (25 ml) and evaporating the solvent slowly at room temperature for about 15 d.

### Refinement

All H atoms were positioned geometrically with C—H = 0.93–0.97 Å and N—H = 0.86 Å, and were and included in the refinement in a riding model approximation, with *U*_iso_(H) = 1.2 or 1.5*U*_eq_(N,*C*).

### Crystal data

**Table:**

C_16_H_18_N_4_O_7_S	*F*_000_ = 1712
*M**_r_* = 410.41	*D*_x_ = 1.505 Mg m^−^^3^
Monoclinic, *C*2/*c*	Melting point = 450–451 K
Hall symbol: -C 2yc	Mo *K*α radiation λ = 0.71073 Å
*a* = 33.831 (7) Å	Cell parameters from 25 reflections
*b* = 6.9020 (14) Å	θ = 10–13º
*c* = 16.021 (3) Å	µ = 0.23 mm^−^^1^
β = 104.48 (3)º	*T* = 298 (2) K
*V* = 3622.1 (13) Å^3^	Needle, colorless
*Z* = 8	0.40 × 0.20 × 0.10 mm

### Data collection

**Table:**

Enraf–Nonius CAD-4 diffractometer	*R*_int_ = 0.035
Radiation source: fine-focus sealed tube	θ_max_ = 25.2º
Monochromator: graphite	θ_min_ = 1.2º
*T* = 298(2) K	*h* = −40→38
ω/2θ scans	*k* = 0→8
Absorption correction: ψ scan(North *et al.*, 1968)	*l* = 0→19
*T*_min_ = 0.914, *T*_max_ = 0.978	3 standard reflections
3325 measured reflections	every 200 reflections
3265 independent reflections	intensity decay: none
2421 reflections with *I* > 2σ(*I*)	

### Refinement

**Table:**

Refinement on *F*^2^	Secondary atom site location: difference Fourier map
Least-squares matrix: full	Hydrogen site location: inferred from neighbouring sites
*R*[*F*^2^ > 2σ(*F*^2^)] = 0.056	H-atom parameters constrained
*wR*(*F*^2^) = 0.156	*w* = 1/[σ^2^(*F*_o_^2^) + (0.08*P*)^2^ + 5*P*] where *P* = (*F*_o_^2^ + 2*F*_c_^2^)/3
*S* = 1.03	(Δ/σ)_max_ < 0.001
3265 reflections	Δρ_max_ = 0.26 e Å^−^^3^
253 parameters	Δρ_min_ = −0.31 e Å^−^^3^
Primary atom site location: structure-invariant direct methods	Extinction correction: none

### Special details

**Table:**

Geometry. All e.s.d.'s (except the e.s.d. in the dihedral angle between two l.s. planes) are estimated using the full covariance matrix. The cell e.s.d.'s are taken into account individually in the estimation of e.s.d.'s in distances, angles and torsion angles; correlations between e.s.d.'s in cell parameters are only used when they are defined by crystal symmetry. An approximate (isotropic) treatment of cell e.s.d.'s is used for estimating e.s.d.'s involving l.s. planes.
Refinement. Refinement of *F*^2^ against ALL reflections. The weighted *R*-factor *wR* and goodness of fit *S* are based on *F*^2^, conventional *R*-factors *R* are based on *F*, with *F* set to zero for negative *F*^2^. The threshold expression of *F*^2^ > σ(*F*^2^) is used only for calculating *R*-factors(gt) *etc*. and is not relevant to the choice of reflections for refinement. *R*-factors based on *F*^2^ are statistically about twice as large as those based on *F*, and *R*- factors based on ALL data will be even larger.

### Fractional atomic coordinates and isotropic or equivalent isotropic displacement parameters (Å^2^)

**Table:**

	*x*	*y*	*z*	*U*_iso_*/*U*_eq_	
S	0.12664 (2)	0.14338 (13)	0.69392 (5)	0.0401 (2)	
N1	0.07804 (7)	0.1705 (4)	0.64572 (16)	0.0424 (7)	
H1A	0.0719	0.1836	0.5906	0.051*	
O1	0.15578 (7)	0.0798 (4)	0.92720 (16)	0.0557 (7)	
C1	0.19376 (12)	−0.2563 (5)	0.9629 (3)	0.0570 (10)	
H1B	0.2121	−0.3646	0.9704	0.085*	
H1C	0.1676	−0.2946	0.9278	0.085*	
H1D	0.1909	−0.2139	1.0182	0.085*	
O2	0.20987 (7)	−0.0991 (3)	0.92109 (16)	0.0484 (6)	
N2	0.00822 (7)	0.1970 (4)	0.63164 (16)	0.0388 (6)	
H2A	−0.0114	0.1857	0.6565	0.047*	
C2	0.18727 (9)	0.0615 (5)	0.90655 (19)	0.0379 (7)	
N3	0.02372 (7)	0.2300 (4)	0.49733 (16)	0.0363 (6)	
O3	0.13329 (8)	−0.0390 (4)	0.73670 (16)	0.0576 (7)	
C3	0.20669 (9)	0.2172 (5)	0.86550 (18)	0.0356 (7)	
O4	0.14650 (7)	0.1838 (4)	0.62677 (15)	0.0542 (7)	
N4	−0.04352 (7)	0.2765 (4)	0.51521 (16)	0.0375 (6)	
C4	0.24906 (9)	0.2405 (5)	0.8922 (2)	0.0432 (8)	
H4C	0.2647	0.1513	0.9300	0.052*	
O5	0.05149 (7)	0.1570 (4)	0.76305 (14)	0.0509 (6)	
C5	0.26803 (10)	0.3949 (5)	0.8631 (2)	0.0470 (8)	
H5A	0.2962	0.4101	0.8819	0.056*	
O6	0.03580 (7)	0.2645 (4)	0.36258 (15)	0.0531 (6)	
C6	0.24518 (11)	0.5258 (6)	0.8063 (2)	0.0505 (9)	
H6A	0.2578	0.6302	0.7870	0.061*	
O7	−0.09468 (7)	0.3699 (4)	0.39861 (15)	0.0508 (6)	
C7	0.20337 (10)	0.5023 (5)	0.7779 (2)	0.0468 (8)	
H7A	0.1883	0.5896	0.7382	0.056*	
C8	0.18338 (9)	0.3511 (5)	0.80731 (19)	0.0363 (7)	
C9	0.13757 (9)	0.3323 (5)	0.7722 (2)	0.0406 (8)	
H9A	0.1264	0.4537	0.7460	0.049*	
H9B	0.1249	0.3034	0.8188	0.049*	
C10	0.04638 (9)	0.1743 (5)	0.6850 (2)	0.0369 (7)	
C11	−0.00401 (9)	0.2356 (4)	0.54321 (19)	0.0341 (7)	
C12	0.00998 (10)	0.2704 (5)	0.4133 (2)	0.0388 (7)	
C13	−0.03018 (10)	0.3179 (5)	0.3764 (2)	0.0438 (8)	
H13A	−0.0395	0.3469	0.3181	0.053*	
C14	−0.05548 (9)	0.3198 (5)	0.4314 (2)	0.0391 (7)	
C15	−0.12294 (10)	0.3408 (6)	0.4504 (2)	0.0573 (10)	
H15A	−0.1496	0.3834	0.4193	0.086*	
H15B	−0.1143	0.4138	0.5028	0.086*	
H15C	−0.1239	0.2056	0.4639	0.086*	
C16	0.07680 (10)	0.1963 (6)	0.3980 (2)	0.0597 (10)	
H16A	0.0918	0.2013	0.3545	0.090*	
H16B	0.0760	0.0652	0.4175	0.090*	
H16C	0.0899	0.2770	0.4457	0.090*	

### Atomic displacement parameters (Å^2^)

**Table:**

	*U*^11^	*U*^22^	*U*^33^	*U*^12^	*U*^13^	*U*^23^
S	0.0287 (4)	0.0485 (5)	0.0398 (4)	0.0025 (3)	0.0022 (3)	−0.0034 (4)
N1	0.0293 (13)	0.0636 (19)	0.0332 (14)	−0.0023 (13)	0.0057 (11)	0.0033 (13)
O1	0.0422 (14)	0.0684 (17)	0.0634 (15)	0.0099 (12)	0.0260 (12)	0.0167 (13)
C1	0.067 (2)	0.041 (2)	0.065 (2)	−0.0069 (18)	0.0199 (19)	0.0080 (19)
O2	0.0434 (13)	0.0434 (14)	0.0610 (15)	0.0055 (10)	0.0178 (11)	0.0100 (11)
N2	0.0292 (13)	0.0450 (16)	0.0394 (14)	0.0004 (11)	0.0035 (11)	0.0083 (12)
C2	0.0350 (17)	0.0447 (18)	0.0313 (15)	0.0025 (14)	0.0032 (13)	−0.0006 (14)
N3	0.0344 (14)	0.0381 (15)	0.0353 (14)	−0.0032 (11)	0.0069 (11)	−0.0003 (11)
O3	0.0556 (16)	0.0440 (15)	0.0620 (16)	0.0068 (12)	−0.0061 (12)	0.0009 (12)
C3	0.0359 (16)	0.0420 (18)	0.0281 (14)	−0.0003 (14)	0.0067 (12)	−0.0035 (13)
O4	0.0377 (13)	0.0835 (19)	0.0434 (13)	−0.0003 (12)	0.0140 (10)	−0.0076 (12)
N4	0.0319 (13)	0.0339 (14)	0.0419 (15)	−0.0018 (11)	0.0001 (11)	0.0019 (12)
C4	0.0339 (16)	0.055 (2)	0.0373 (17)	0.0008 (15)	0.0018 (13)	−0.0002 (16)
O5	0.0351 (12)	0.0769 (18)	0.0383 (13)	0.0013 (12)	0.0046 (10)	0.0061 (12)
C5	0.0336 (17)	0.062 (2)	0.0423 (18)	−0.0094 (16)	0.0041 (14)	−0.0014 (17)
O6	0.0460 (14)	0.0707 (17)	0.0428 (13)	0.0023 (12)	0.0117 (11)	0.0010 (12)
C6	0.049 (2)	0.057 (2)	0.0463 (19)	−0.0161 (17)	0.0120 (16)	0.0005 (17)
O7	0.0359 (12)	0.0585 (15)	0.0513 (14)	0.0048 (11)	−0.0016 (10)	0.0134 (12)
C7	0.0469 (19)	0.050 (2)	0.0393 (17)	0.0003 (16)	0.0035 (15)	0.0036 (16)
C8	0.0306 (15)	0.0397 (17)	0.0360 (16)	−0.0009 (13)	0.0035 (12)	−0.0060 (14)
C9	0.0344 (16)	0.0445 (19)	0.0406 (17)	0.0049 (14)	0.0050 (13)	−0.0014 (15)
C10	0.0304 (15)	0.0371 (17)	0.0412 (18)	−0.0007 (13)	0.0052 (13)	0.0015 (14)
C11	0.0312 (15)	0.0291 (16)	0.0389 (16)	−0.0024 (12)	0.0028 (13)	0.0013 (13)
C12	0.0439 (18)	0.0330 (17)	0.0385 (17)	−0.0033 (14)	0.0083 (14)	−0.0030 (14)
C13	0.0460 (19)	0.045 (2)	0.0340 (16)	−0.0047 (15)	−0.0020 (14)	0.0006 (14)
C14	0.0363 (17)	0.0323 (17)	0.0433 (18)	−0.0011 (13)	−0.0001 (14)	0.0054 (14)
C15	0.0384 (19)	0.068 (3)	0.062 (2)	0.0021 (18)	0.0057 (17)	0.011 (2)
C16	0.0396 (19)	0.081 (3)	0.057 (2)	−0.0040 (19)	0.0105 (16)	−0.009 (2)

### Geometric parameters (Å, °)

**Table:**

S—O3	1.424 (3)	O5—C10	1.224 (4)
S—O4	1.431 (2)	C5—C6	1.374 (5)
S—N1	1.643 (3)	C5—H5A	0.930
S—C9	1.782 (3)	O6—C12	1.334 (4)
N1—C10	1.372 (4)	O6—C16	1.440 (4)
N1—H1A	0.860	C6—C7	1.383 (5)
O1—C2	1.199 (4)	C6—H6A	0.930
C1—O2	1.451 (4)	O7—C14	1.344 (4)
C1—H1B	0.960	O7—C15	1.428 (4)
C1—H1C	0.960	C7—C8	1.388 (5)
C1—H1D	0.960	C7—H7A	0.930
O2—C2	1.333 (4)	C8—C9	1.516 (4)
N2—C10	1.368 (4)	C9—H9A	0.970
N2—C11	1.399 (4)	C9—H9B	0.970
N2—H2A	0.860	C12—C13	1.379 (4)
C2—C3	1.495 (5)	C13—C14	1.373 (5)
N3—C11	1.329 (4)	C13—H13A	0.930
N3—C12	1.339 (4)	C15—H15A	0.960
C3—C4	1.399 (4)	C15—H15B	0.960
C3—C8	1.406 (4)	C15—H15C	0.960
N4—C11	1.330 (4)	C16—H16A	0.960
N4—C14	1.336 (4)	C16—H16B	0.960
C4—C5	1.384 (5)	C16—H16C	0.960
C4—H4C	0.930		
			
O3—S—O4	119.13 (17)	C6—C7—H7A	119.3
O3—S—N1	110.25 (15)	C8—C7—H7A	119.3
O4—S—N1	103.13 (14)	C7—C8—C3	118.6 (3)
O3—S—C9	109.17 (16)	C7—C8—C9	118.5 (3)
O4—S—C9	109.40 (16)	C3—C8—C9	122.8 (3)
N1—S—C9	104.73 (15)	C8—C9—S	109.7 (2)
C10—N1—S	126.2 (2)	C8—C9—H9A	109.7
C10—N1—H1A	116.9	S—C9—H9A	109.7
S—N1—H1A	116.9	C8—C9—H9B	109.7
O2—C1—H1B	109.5	S—C9—H9B	109.7
O2—C1—H1C	109.5	H9A—C9—H9B	108.2
H1B—C1—H1C	109.5	O5—C10—N2	121.3 (3)
O2—C1—H1D	109.5	O5—C10—N1	122.7 (3)
H1B—C1—H1D	109.5	N2—C10—N1	116.0 (3)
H1C—C1—H1D	109.5	N3—C11—N4	127.5 (3)
C2—O2—C1	115.9 (3)	N3—C11—N2	118.9 (3)
C10—N2—C11	130.5 (3)	N4—C11—N2	113.6 (3)
C10—N2—H2A	114.8	O6—C12—N3	119.4 (3)
C11—N2—H2A	114.8	O6—C12—C13	118.1 (3)
O1—C2—O2	123.4 (3)	N3—C12—C13	122.5 (3)
O1—C2—C3	124.3 (3)	C14—C13—C12	115.6 (3)
O2—C2—C3	112.2 (3)	C14—C13—H13A	122.2
C11—N3—C12	115.7 (3)	C12—C13—H13A	122.2
C4—C3—C8	119.3 (3)	N4—C14—O7	118.1 (3)
C4—C3—C2	118.5 (3)	N4—C14—C13	124.2 (3)
C8—C3—C2	121.9 (3)	O7—C14—C13	117.7 (3)
C11—N4—C14	114.4 (3)	O7—C15—H15A	109.5
C5—C4—C3	120.8 (3)	O7—C15—H15B	109.5
C5—C4—H4C	119.6	H15A—C15—H15B	109.5
C3—C4—H4C	119.6	O7—C15—H15C	109.5
C6—C5—C4	119.8 (3)	H15A—C15—H15C	109.5
C6—C5—H5A	120.1	H15B—C15—H15C	109.5
C4—C5—H5A	120.1	O6—C16—H16A	109.5
C12—O6—C16	118.8 (3)	O6—C16—H16B	109.5
C5—C6—C7	120.1 (3)	H16A—C16—H16B	109.5
C5—C6—H6A	120.0	O6—C16—H16C	109.5
C7—C6—H6A	120.0	H16A—C16—H16C	109.5
C14—O7—C15	118.4 (3)	H16B—C16—H16C	109.5
C6—C7—C8	121.4 (3)		
			
O3—S—N1—C10	61.5 (3)	N1—S—C9—C8	−167.4 (2)
O4—S—N1—C10	−170.2 (3)	C11—N2—C10—O5	173.7 (3)
C9—S—N1—C10	−55.8 (3)	C11—N2—C10—N1	−6.9 (5)
C1—O2—C2—O1	1.1 (5)	S—N1—C10—O5	−0.1 (5)
C1—O2—C2—C3	178.5 (3)	S—N1—C10—N2	−179.5 (2)
O1—C2—C3—C4	139.0 (3)	C12—N3—C11—N4	0.0 (5)
O2—C2—C3—C4	−38.4 (4)	C12—N3—C11—N2	−179.3 (3)
O1—C2—C3—C8	−34.9 (5)	C14—N4—C11—N3	−1.4 (5)
O2—C2—C3—C8	147.7 (3)	C14—N4—C11—N2	178.0 (3)
C8—C3—C4—C5	1.0 (5)	C10—N2—C11—N3	8.8 (5)
C2—C3—C4—C5	−173.1 (3)	C10—N2—C11—N4	−170.6 (3)
C3—C4—C5—C6	−0.9 (5)	C16—O6—C12—N3	6.0 (5)
C4—C5—C6—C7	−0.5 (5)	C16—O6—C12—C13	−173.8 (3)
C5—C6—C7—C8	1.9 (5)	C11—N3—C12—O6	−178.8 (3)
C6—C7—C8—C3	−1.7 (5)	C11—N3—C12—C13	1.0 (5)
C6—C7—C8—C9	−179.0 (3)	O6—C12—C13—C14	179.3 (3)
C4—C3—C8—C7	0.3 (4)	N3—C12—C13—C14	−0.5 (5)
C2—C3—C8—C7	174.2 (3)	C11—N4—C14—O7	−177.5 (3)
C4—C3—C8—C9	177.5 (3)	C11—N4—C14—C13	1.9 (5)
C2—C3—C8—C9	−8.7 (5)	C15—O7—C14—N4	−11.2 (4)
C7—C8—C9—S	102.8 (3)	C15—O7—C14—C13	169.4 (3)
C3—C8—C9—S	−74.3 (3)	C12—C13—C14—N4	−1.0 (5)
O3—S—C9—C8	74.5 (3)	C12—C13—C14—O7	178.4 (3)
O4—S—C9—C8	−57.5 (3)		

### Hydrogen-bond geometry (Å, °)

**Table:**

*D*—H···*A*	*D*—H	H···*A*	*D*···*A*	*D*—H···*A*
N1—H1A···N3	0.86	1.94	2.648 (4)	138
N2—H2A···O5^i^	0.86	2.10	2.951 (3)	170
C9—H9B···O1	0.97	2.36	2.970 (4)	120
C15—H15C···O1^i^	0.96	2.43	3.068 (4)	124

Symmetry codes: (i) −*x*, *y*, −*z*+3/2.
